# MultiSeq: unifying sequence and structure data for evolutionary analysis

**DOI:** 10.1186/1471-2105-7-382

**Published:** 2006-08-16

**Authors:** Elijah Roberts, John Eargle, Dan Wright, Zaida Luthey-Schulten

**Affiliations:** 1 Center for Biophysics and Computational Biology, University of Illinois at Urbana-Champaign, Urbana, IL, USA; 2 Graduate School of Library and Information Sciences, University of Illinois at Urbana-Champaign, Urbana, IL, USA; 3 Department of Chemistry, University of Illinois at Urbana-Champaign, Urbana, IL, USA

## Abstract

**Background:**

Since the publication of the first draft of the human genome in 2000, bioinformatic data have been accumulating at an overwhelming pace. Currently, more than 3 million sequences and 35 thousand structures of proteins and nucleic acids are available in public databases. Finding correlations in and between these data to answer critical research questions is extremely challenging. This problem needs to be approached from several directions: information science to organize and search the data; information visualization to assist in recognizing correlations; mathematics to formulate statistical inferences; and biology to analyze chemical and physical properties in terms of sequence and structure changes.

**Results:**

Here we present MultiSeq, a unified bioinformatics analysis environment that allows one to organize, display, align and analyze both sequence and structure data for proteins and nucleic acids. While special emphasis is placed on analyzing the data within the framework of evolutionary biology, the environment is also flexible enough to accommodate other usage patterns. The evolutionary approach is supported by the use of predefined metadata, adherence to standard ontological mappings, and the ability for the user to adjust these classifications using an electronic notebook. MultiSeq contains a new algorithm to generate complete evolutionary profiles that represent the topology of the molecular phylogenetic tree of a homologous group of distantly related proteins. The method, based on the multidimensional QR factorization of multiple sequence and structure alignments, removes redundancy from the alignments and orders the protein sequences by increasing linear dependence, resulting in the identification of a minimal basis set of sequences that spans the evolutionary space of the homologous group of proteins.

**Conclusion:**

MultiSeq is a major extension of the Multiple Alignment tool that is provided as part of VMD, a structural visualization program for analyzing molecular dynamics simulations. Both are freely distributed by the NIH Resource for Macromolecular Modeling and Bioinformatics and MultiSeq is included with VMD starting with version 1.8.5. The MultiSeq website has details on how to download and use the software:

## Background

In the field of bioinformatics, research activities are often split into two distinct areas: sequence analysis or structure analysis. Genomic and other sequencing projects generate enormous amounts of sequence data that are initially released with large portions annotated as either putative or hypothetical. Structural data, even in the era of structural genomics, are produced at a slower pace but analyzed to a high degree before being deposited into the public databases, such as PDB [1], SCOP [2,3]/Astral [4], CATH [5] and NDB [6]. This difference in pace has led to an increasing discrepancy in the relative sizes of these two data sets. The total size of the sequence databases (NCBI [7,8], EMBL [9], DDBJ [10], JGI [11], RDP [12], Swiss-Prot/TrEMBL [13], CRW [14], Bayreuth tRNA compilations [15], and the Genomic tRNA Database [16]) is several orders of magnitude greater than that of the structure databases. For a given protein, the large number of available sequences allows more complete evolutionary analyses. Multiple sequence alignments (MSA) are instrumental in identifying key conserved areas of a sequence, developing an evolutionary history of a molecule [17], and examining the covariance within a sequence in response to evolutionary pressure [18]. These analyses depend on having enough sequences to perform a well-balanced statistical analysis. The advantages of structural data are that they provide much more detailed information about the molecule in question, allowing specific atomic level interactions to be analyzed. Additionally, since structure is more conserved than sequence [19], structural data can be used to reconstruct many of the deeper evolutionary branches that would be difficult or impossible to determine with sequence data alone [20-23].

VMD [24], which currently has more than 30,000 registered users, provides powerful visualization and analysis capabilities for both structural and dynamics data generated from molecular dynamics simulations, as well as energetics derived from molecular mechanics force fields. It is optimized to handle large scale systems containing millions of atoms. VMD also implements a flexible scripting interface for the creation of custom tools. The previous Multiple Alignment [25] extension to VMD added only the capability to use evolutionary information obtained from multiple structures for interpreting structural results. Our goal with MultiSeq is to extend VMD's capabilities further by incorporating the more diverse evolutionary data available in sequences into the analysis process.

There are already a large number of tools available to analyze bioinformatic data, but, like the field itself, they are mostly segregated into either sequence or structure tools. In the sequence world there are tools for viewing, analyzing, and editing an MSA like AE2 [26], CINEMA [27], ClustalX [28], and Jalview [29]; there are tools for creating MSAs by aligning individual sequences and profiles, like ClustalW [30], HMMER [31] and T-Coffee [32]; there are tools for annotating sequence data such as Pfaat [33]; and BLAST [34] is used for searching through databases for related sequences. One popular package, MEGA3 [35], provides an evolutionary approach to analyzing protein and nucleic acid sequences, including many easy to use features for determining sequence based phylogenies. Similarly, in the structure world there are numerous tools for visualizing structural data and performing structural analyses, including RASMOL [36], STAMP [37], STRIDE [38], and 3DNA [39].

There are also a few programs that combine sequence and structure data either for specific purposes, such as Swiss-PdbViewer/SWISS-MODEL [40] and MolIDE/SCWRL3 [41,42] for homology modeling, or as part of a pre-computed database of attributes, of which STING [43] is the primary example. Modeller [44] allows structural features to be built using sequences and structures of homologous proteins and nucleic acids. UCSF Chimera [45], a well known molecular modeling program that originated to handle small molecule docking, provides the ability to use sequence data in conjunction with structural data. However, it lacks some of the features needed to perform well-balanced evolutionary analyses, such as phylogenetic tree construction and elimination of bias. Friend [46], a bioinformatics application, has many of the sequence features required for performing evolutionary analyses, but has insufficient structural functionality to fully interpret the results in a structural context. InsightII and Discovery Studio (Accelrys Inc.) and MOE (Chemical Computing Group Inc.) are popular commercial packages for analyzing protein/drug interactions based on protein structure, dynamics, and energetics. Both programs can also use sequence data to perform combined analyses. NCBI's Cn3D [47] also supports both sequence and structure data, although it is primarily designed for use with pre-computed 3D superpositions and MSAs.

The complementary information provided by fusion of these two data sources can give insight into evolutionary changes in sequence, structure, and function. However, the conceptual spaces of these fields differ, often resulting in mutual incomprehensibility to researchers in each field. MultiSeq, in dealing with both sequence and structure data in a way that is accessible to both areas, helps to bridge this gap. It does so through the use of integrated cross-referencing that acts as an informal version of ontology-driven knowledge extraction and discovery [48]. We plan to enhance future versions of MultiSeq to incorporate formal ontological methods, including using the work of groups such as the Gene Ontology project [49].

There is tremendous utility to be had in combining both sequence and structure data within an evolutionary framework using the four pillars of information science, information visualization, mathematics, and biology to organize the flow of information. An evolutionary profile (EP) is a concise and complete representation of the diversity that has been generated by the evolutionary process within a homologous group of proteins. A key step in the creation of an EP is the elimination of redundancy present in the sequence and structural databases [50] due to bias in the selection of organisms chosen for study. The sequence and structure QR algorithms have been developed specifically to address this problem [21,22]. These smaller, more evolutionarily balanced profiles have comparable, and in many cases better, performance in database searches than conventional profiles containing hundreds of sequences. For more diverse families or superfamilies, with sequence identity < 30%, structural alignments, based purely on the geometry of the protein structures, provide better alignments than pure sequence-based methods. Merging the structure and sequence information allows the construction of accurate profiles for distantly related groups. The success of using sequence and structure based EPs for both gene annotation [23] and the prediction of structurally conserved motifs [51] shows their effectiveness. We also anticipate the usefulness of EPs for studying, among other things, the relationship between protein structure and stability, the evolution of protein/RNA interfaces, and the basis of protein conformational motion.

The actual process of creating an EP is detailed in Sethi et. al. [22], a tutorial [52], and a forthcoming applications paper, but can be summarized as follows:

1. Load a set of sequences and structures and their associated metadata.

2. Align the data, using structural alignments as profiles for aligning widely divergent sequence groups.

3. Perform a phylogenetic analysis to determine the evolutionary relationships in the data.

4. Check and adjust the alignment using the phylogenetic tree and taxonomic information as guides.

5. Eliminate any redundant data that may be a source of bias.

**Figure 1:**
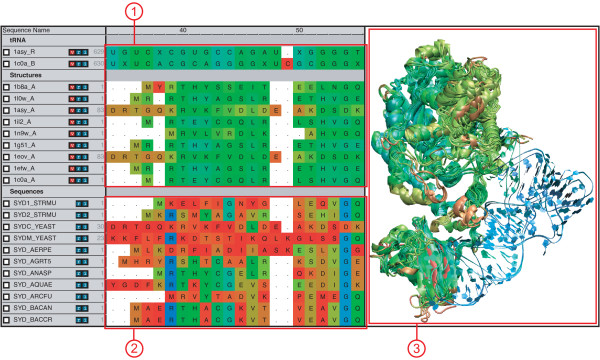
**MultiSeq Overview**. Overview of the MultiSeq environment showing aligned sequence and structural data. (1) 1D representation of structural data colored by structural conservation. (2) 1D representation of sequence data colored by sequence identity. (3) 3D representation of structural data colored by structural conservation, as shown by VMD. For structural data, the coloring is synchronized between the 1D representation and the 3D representation.

Within this process there are many difficulties, such as identifying horizontal gene transfer (HGT) events and misannotated data, both important for proper grouping of evolutionary data, and developing a statistically well-balanced set of sequences and structures. MultiSeq attempts to lower some of these barriers to combining sequence and structural data into EPs by consolidating the tools necessary to perform such analyses in an intuitive software package (Figure 1).

## Implementation and discussion

### Importing protein data for analysis

The primary function of MultiSeq is to provide an environment for the evolutionary analysis of bioinformatic data from both structure and sequence. Before any analysis can be performed, however, the data must first be imported into the environment, which is often a non-trivial task given the wide variety of sources from which data may be acquired. MultiSeq provides a consistent interface to allow data from numerous sources to be quickly and easily brought into the environment and consolidated for further analysis.

Structural data for biomolecules come in a bewildering array of file formats, a large number of which can be read and processed by VMD. To take advantage of this capability, MultiSeq relies on VMD to parse structure files and present a 3D representation of the data. After VMD has loaded the structural data, MultiSeq creates a copy of the sequence portion of the data, stores that in its own internal data structures for use when displaying 1D representations of the data (see Figure 1), and then establishes a link between its internal data structures and those of VMD. This synergy means that MultiSeq works with every format of structural data that VMD supports, including such common formats as PDB, XYZ, NetCDF, and CHARMM. MultiSeq also makes it easy to load multiple structures, which is necessary during the construction of a structural profile. Additionally, MultiSeq extends VMD's ability to load protein structures over the Internet by allowing multiple PDB codes to be specified and individual domains of protein structures to be loaded directly from the Astral database [4].

Sequence data are often stored in a single file containing multiple sequences, in either an aligned or an unaligned state. MultiSeq can load sequence files formatted in ALN, FASTA, Nexus, PIR, and PHY file formats. For FASTA formatted files, description lines are preserved and made available through the electronic notebook, described below. Upon loading a sequence file, MultiSeq can automatically download corresponding structural data, if it is available in one of the known structural databases. Currently supported structural databases are the PDB, Astral, and the subset of Swiss-Prot that is derived from the PDB.

**Figure 2:**
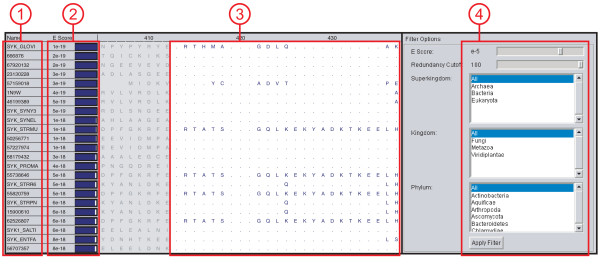
**BLAST Results Viewer**. BLAST search results viewer showing the outcome of a BLAST search. (1) The name of the matching sequence is shown along with (2) the expectation value of the match. (3) The BLAST aligned regions are shown as an MSA; non-matched regions on either side of the aligned region are shown grayed out. (4) The search results can be filtered by BLAST e-score, taxonomy, or sequence QR based redundancy.

A final method of loading protein sequence data into MultiSeq is through the use of a BLASTP [53] search. Given a target sequence or profile, BLAST discovers a variety of homologous sequences which can then be incorporated into the analysis. MultiSeq uses a locally installed version of BLAST to search local sequence databases using a single sequence, a profile of sequences [34], or a fragment of a sequence or profile. The search can be performed a single time or iteratively using PSI-BLAST, and the search results are displayed and filtered before being imported into MultiSeq, as shown in Figure 2. Current filtering options include BLAST e-score, taxonomic classification, and a redundancy filter based on the sequence QR algorithm [22] on the BLAST generated alignment. As when loading sequence data from a file, any corresponding structural data for the search results can be automatically downloaded when the search results are imported. This opens up the possibility of running a BLAST search against the PDB or Astral databases to load structures that share sequence similarity with a source sequence or profile, a feature that is particularly useful for finding a template during homology modeling of a protein of unknown structure [51].

Depending on the size of the protein, MultiSeq can easily load hundreds of sequences and/or structures. The time required to perform an analysis of such a large set, however, depends on the analysis method being used.

### Organizing data to accommodate various analysis frameworks

**Figure 3:**
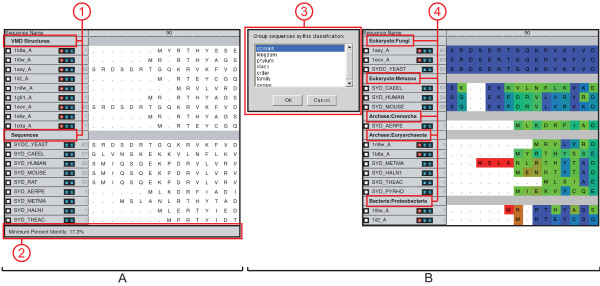
**Grouping**. [A] Grouping in MultiSeq. (1) Group headers show the name of the group and allow the user to manage the group. (2) The status bar shows summary information about the group. [B] MultiSeq allows data to be automatically grouped by taxonomic classification. (3) The taxonomy dialog allows the user to select the level of taxonomy by which to group the data. (4) Taxonomic information about the data is then used to create the groupings.

The number of different sources of sequences and structures can be intimidating and calls for an organizational framework in which to work with the data. At the same time, the varied uses of these data demand that the framework be flexible enough to accommodate a wide variety of users. MultiSeq addresses this issue by implementing a *flexible grouping system*. Each sequence in MultiSeq is displayed beneath its group in the main display, as shown in Figure 3A. The group header acts as an interface anchor to allow the user to perform operations on the group as a whole, and the status bar shows overview information about the currently selected group. The default grouping is based on the source of the data, i.e., structures loaded through VMD appear in the **VMD Structures **group and sequences loaded by a BLAST search appear in the **BLAST Results **group, but the user can easily expand, rename, and reorder these groupings as appropriate for the situation at hand. Additionally, the data can be automatically grouped by taxonomic classifications (Figure 3B). Separating the data into evolutionarily distinct groupings allows any analysis to be easily performed on each related group independently.

### Finding metadata automatically via the internet

Metadata (or "data about data") – such as taxonomy, enzymatic function, or structural classification – related to sequence and structural data can provide valuable insight during many bioinformatic analyses. Various databases accessible via the Internet store this information and present it when displaying results but otherwise make little use of it. MultiSeq correlates this metadata by cross-referencing both the name of the sequence or structure and any source information contained in the original file. Currently, MultiSeq can extract NCBI taxonomy information [54], Enzyme Commission (EC) numbers, and SCOP structural classifications [2]. MultiSeq integrates this metadata into the evolutionary analysis process through grouping and phylogenetic tree functions.

**Figure 4:**
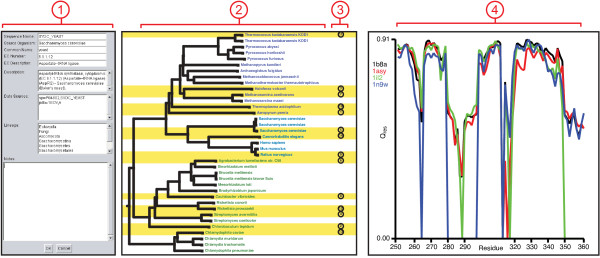
**MultiSeq Tools**. (1) The electronic notebook displays various metadata associated with the sequence and also provides space for making annotations about a sequence. Changes will be saved in the MultiSeq session. (2) The phylogenetic tree viewer shows evolutionary relationships amongst the data. Data are labeled by species name and colored by domain of life, those highlighted in yellow are part of the selected non-redundant set. (3) The QR ordering of the non-redundant set is also displayed, lower numbers indicate data that are more linearly independent. (4) The plotter allows a metric to be plotted along the length (or a subset) of the sequence. All of the coloring metrics can also be used by the plotter.

Metadata can be added, viewed, and edited using the electronic notebook (Figure 4). The electronic notebook provides a consistent way to interact with all available metadata for a sequence, regardless of its source. It also provides a place to store notes regarding the sequence and any processing that has been performed on it. Changes to the metadata are saved along with a MultiSeq session, described below.

### Alignment of sequence and structural data

In order to properly analyze multiple homologous sequences and structures, they must first be aligned. For structural data, a version of STAMP [37] that has been modified to better align end regions (details of the modifications are available in the methods section) is used to perform the alignment. For sequence data ClustalW [30] is used. In the next version of MultiSeq, a plug-in framework is planned to allow other sequence and structural alignment programs, such as HMMER [31] and T-Coffee [32], to be used. It is generally accepted that structural alignments are more reliable than sequence alignments for distantly related proteins and RNA molecules [55,56,19], so MultiSeq allows a structural alignment to be passed to ClustalW as a profile to seed the alignment process. This technique can be particularly effective when the structural profile is in fact an evolutionary profile and contains a non-redundant sampling of distantly related structures. In either case, though, the alignment is rarely perfect and some manual editing is usually required using MultiSeq's built-in editor.

### QR algorithms to eliminate redundancy and bias in data

Although the vast quantity of data available in this post-genomic era brings many new possibilities for analysis, it also opens up the potential for introducing systematic errors in these analyses due to the biases inherent in the makeup of the various databases. MultiSeq includes both the sequence QR [22] and structure QR [21] algorithms to help detect and eliminate this redundancy during any step of the analysis process. The sequence and structure QR algorithms orthogonally encode a multiple sequence or structure alignment as a multidimensional matrix, and then perform a QR factorization on this matrix [57]. The result is an ordering of the sequences or structures from most linearly independent to least independent. A non-redundant set from amongst the available data (See Figure 4) is constructed by specifying a cutoff in either sequence or structural similarity. The QR algorithms can also be run on a specific region of the MSA so that the non-redundant set can be generated based on, for example, one domain of a multi-domain protein or an insertion in the sequence. The sequence and structure QR algorithms combined with the grouping and selection capabilities of MultiSeq constitute a powerful environment for constructing EPs for use in bioinformatics-intensive tasks such as homology modeling [51] or gene annotation [22,23].

### Analyzing phylogenetic relationships

Phylogenetic trees, which show the relationships between related proteins or nucleic acids, are invaluable when performing evolutionary analyses. They provide a guide for investigating why and how certain attributes developed as well as identifying misalignments and HGT events. The accuracy and speed of various tree reconstruction methods, however, varies widely from simple distance based methods such as unweighted pair group method with arithmetic averages (UPGMA) [58] and neighbor-joining (NJ) to complex methods such as maximum likelihood [59], which take into account an underlying theory of evolution. In general, distance based trees are sufficient for many common uses [60]. MultiSeq creates UPGMA trees using the structural measures Q_H _[20] and root mean square deviation (RMSD) as well as the sequence measure of percent identity. It also creates trees based on similarity using the NJ method of CLUSTALW [30]. After a tree has been computed, it can be decorated and colored with various attributes such as species name, domain of life, and enzymatic function, as shown in Figure 4. Additionally, various manipulations such as collapsing, rotating, and labeling nodes to assist in visualization can be performed.

One further use for phylogenetic trees within MultiSeq is in conjunction with the QR algorithms to eliminate redundancy from data. When either the SeqQR or StructQR tools are used on data being displayed in a phylogenetic tree, those data are highlighted both within the main environment and within the tree viewer (Figure 4). This feature allows for evaluation of the non-redundant selection so that the user can adjust the cutoff. The orderings from the QR algorithm indicating which data are most linearly independent are also displayed in the tree to assist in this process.

### Using visualization to illuminate trends

One way to effectively present complex information is to color code the data using attributes that are not normally visible [61]. MultiSeq presets attributes of the data as coloring in both the 1D representation of the sequence portion of the data and the 3D representation of the structural portion of the data. It maintains a consistent coloring between the two representations in order to facilitate an easy mental transition between them. Many different sequence and structural metrics are currently implemented as coloring choices and the addition of custom coloring methods is supported through a programming interface. The current list of standard metrics is: sequence conservation, sequence entropy, percent sequence identity, sequence similarity, Q_res _structural similarity, residue type, and structural RMSD. In addition to calculating attribute values, MultiSeq can import them from a tab or space delimited file. This enables the importing of other types of attribute data, such as those from HD exchange or Φ-value experiments.

Many of the above coloring metrics are calculated by comparing two or more sequences or structures to get a value representing the specified attribute. The default behavior is to use all of the loaded data in the calculation of a metric, however, one can optionally have MultiSeq process each group independently. Using this feature one can, for example, view sequence identity across all domains of life and then quickly switch to sequence identity within the individual domains. This capability can be very useful for identifying a signature of a specific group of sequences or structures.

Another method of assisting in visualization is to hide attributes that provide no relevant information in the current context. Often, eliminating this extraneous information can lead to patterns being more quickly understood. One common technique of dealing with this issue in the world of structural biology is through the use of secondary structure representations, showing only the backbone and secondary structure elements of a protein. VMD supports secondary structure, as calculated by STRIDE [38], as a 3D representation and MultiSeq can display it for structures as a graphical 1D representation. MultiSeq also provides a bar and line representation that is particularly useful when visualizing attributes that are zero over portions of the sequence, such as experimental data.

### Nucleic acid sequences and structures

MultiSeq also supports bioinformatic analysis of both nucleic acid sequence and structure data, but the tools are somewhat more limited in the present release. Nucleic acid sequences may be imported as unaligned sequences or as MSAs using any of file formats supported for protein sequences. These data may be obtained from a variety of databases including IMG, NCBI (Genbank), Bayreuth tRNA compilations, CRW, RDP, and the Genomic tRNA Database. We provide external scripts to convert files from AE2 format (provided by Gary Olsen) and Bayreuth flatfiles to the FASTA format [62]. BLASTN support for finding related nucleic acid sequences is planned for the next release.

Once nucleic acid sequence data have been loaded, multiple sequence alignments can be computed using the ClustalW interface within MultiSeq. Only coloring by sequence identity works with nucleic acids, other sequence-based coloring metrics specific for nucleic acid will be incorporated in the next release. STAMP has been modified to align nucleic acid structures by their backbone phosphorous atoms resulting in a structural alignment analogous to *α*-carbon based alignment for proteins. When the alignment is complete the 3D representation displays the structural superposition of the aligned molecules. The built-in structural alignment analysis tools, such as structure-based trees and coloring metrics, work correctly with nucleic acid structural alignments.

RNA molecules frequently incorporate nonstandard modified nucleotides that can affect folding, structure, and function. For example, the T*ψ*C loop in tRNA typically contains a *ψ*, or pseudouridine, base. There are on the order of 100 RNA-associated modified bases identified at this time [63]. The RNA molecule, as opposed to its DNA gene, must be sequenced to determine the modified bases included. When this information is available in structure or sequence files MultiSeq recognizes and appropriately displays modified bases in the 1D representation. In the next release of MultiSeq, QR will be available for nucleic acids, and a canonical, evolutionarily balanced 16S rRNA will be incorporated to help with phylogenetic analysis. At that time secondary structure analysis tools for nucleic acid structures will also be included.

### Exporting data

It is often desirable to preserve an entire bioinformatic analysis so that work can be resumed at a later time. MultiSeq implements this by saving the entire environment as a session. When a session is saved, all of the sequence and structure data loaded into VMD and MultiSeq are saved along with any alignments and transformations that have been applied to them. Metrics, annotations, metadata changes, and representation choices are also saved with the session. Once a session has been saved, it can later be loaded and work resumed quickly and easily.

MultiSeq also supports numerous formats for exporting all or a subset of the data in the environment to a file. This can be useful if an analysis needs to be run using external bioinformatics software. For example, MultiSeq can export all of the files necessary to run a maximum likelihood/parsimony based phylogenetic analysis of sequence data using PAUP* [64], PHYLIP [65], and PHYML [66] or a Bayesian based analysis using MrBayes [67].

A final feature of note is MultiSeq's ability to export publication quality graphics. The sequence window, tree viewer, and plotter can all save PostScript files of their current representation. Since these are vector graphics, they can be scaled and manipulated using illustration software with no loss of quality.

## Methods

**Q**_res _– We use a measure called Q_res _to calculate structural similarity of each residue in a set of aligned structures. It is derived from Q, which is used in protein folding to compare the pair distances in a protein conformation to the native one [68]. We have previously used this measure for deriving protein cores by looking at structural conservation [21,20]. Q_res _computes the similarity of the C_*α*_-C_*α *_distances between a residue and all other residues in the protein, excluding nearest neighbors, to the corresponding distances in a given set of proteins. The result is a value between 0 and 1 that describes the similarity of the structural environment of a residue in a particular protein to the environment of that same residue in all other proteins in the set. Lower scores indicate low similarity and higher scores high similarity. Formally, Q_res _is defined as follows:

Qres(i,n)=ℵ∑(m≠n)proteins∑(j≠i−1,i,i+1)residuesexp⁡[−(rij(n)−ri′j′(m))22σij2]     (1)
 MathType@MTEF@5@5@+=feaafiart1ev1aaatCvAUfKttLearuWrP9MDH5MBPbIqV92AaeXatLxBI9gBaebbnrfifHhDYfgasaacH8akY=wiFfYdH8Gipec8Eeeu0xXdbba9frFj0=OqFfea0dXdd9vqai=hGuQ8kuc9pgc9s8qqaq=dirpe0xb9q8qiLsFr0=vr0=vr0dc8meaabaqaciaacaGaaeqabaqabeGadaaakeaacqWGrbqudaqhaaWcbaGaemOCaiNaemyzauMaem4CamhabaGaeiikaGIaemyAaKMaeiilaWIaemOBa4MaeiykaKcaaOGaeyypa0JaeyynHa8aaabCaeaadaaeWbqaaiGbcwgaLjabcIha4jabcchaWnaadmGabaGaeyOeI0YaaSaaaeaadaqadiqaaiabdkhaYnaaDaaaleaacqWGPbqAcqWGQbGAaeaacqGGOaakcqWGUbGBcqGGPaqkaaGccqGHsislcqWGYbGCdaqhaaWcbaGafmyAaKMbauaacuWGQbGAgaqbaaqaaiabcIcaOiabd2gaTjabcMcaPaaaaOGaayjkaiaawMcaamaaCaaaleqabaGaeGOmaidaaaGcbaGaeGOmaidcciGae83Wdm3aa0baaSqaaiabdMgaPjabdQgaQbqaaiabikdaYaaaaaaakiaawUfacaGLDbaaaSqaaiabcIcaOiabdQgaQjabgcMi5kabdMgaPjabgkHiTiabigdaXiabcYcaSiabdMgaPjabcYcaSiabdMgaPjabgUcaRiabigdaXiabcMcaPaqaaiabdkhaYjabdwgaLjabdohaZjabdMgaPjabdsgaKjabdwha1jabdwgaLjabdohaZbqdcqGHris5aaWcbaGaeiikaGIaemyBa0MaeyiyIKRaemOBa4MaeiykaKcabaGaemiCaaNaemOCaiNaem4Ba8MaemiDaqNaemyzauMaemyAaKMaemOBa4Maem4CamhaniabggHiLdGccaWLjaGaaCzcamaabmGabaGaeGymaedacaGLOaGaayzkaaaaaa@8DBA@

where Qres(i,n)
 MathType@MTEF@5@5@+=feaafiart1ev1aaatCvAUfKttLearuWrP9MDH5MBPbIqV92AaeXatLxBI9gBaebbnrfifHhDYfgasaacH8akY=wiFfYdH8Gipec8Eeeu0xXdbba9frFj0=OqFfea0dXdd9vqai=hGuQ8kuc9pgc9s8qqaq=dirpe0xb9q8qiLsFr0=vr0=vr0dc8meaabaqaciaacaGaaeqabaqabeGadaaakeaacqWGrbqudaqhaaWcbaGaemOCaiNaemyzauMaem4CamhabaGaeiikaGIaemyAaKMaeiilaWIaemOBa4MaeiykaKcaaaaa@3785@ is the structural similarity of the *i*^*th *^residue in the *n*^*th *^protein, rij(n)
 MathType@MTEF@5@5@+=feaafiart1ev1aaatCvAUfKttLearuWrP9MDH5MBPbIqV92AaeXatLxBI9gBaebbnrfifHhDYfgasaacH8akY=wiFfYdH8Gipec8Eeeu0xXdbba9frFj0=OqFfea0dXdd9vqai=hGuQ8kuc9pgc9s8qqaq=dirpe0xb9q8qiLsFr0=vr0=vr0dc8meaabaqaciaacaGaaeqabaqabeGadaaakeaacqWGYbGCdaqhaaWcbaGaemyAaKMaemOAaOgabaGaeiikaGIaemOBa4MaeiykaKcaaaaa@3415@ is the *C*_*α*_-*C*_*α *_distance between residues *i *and *j *in protein *n *and ri′j′(m)
 MathType@MTEF@5@5@+=feaafiart1ev1aaatCvAUfKttLearuWrP9MDH5MBPbIqV92AaeXatLxBI9gBaebbnrfifHhDYfgasaacH8akY=wiFfYdH8Gipec8Eeeu0xXdbba9frFj0=OqFfea0dXdd9vqai=hGuQ8kuc9pgc9s8qqaq=dirpe0xb9q8qiLsFr0=vr0=vr0dc8meaabaqaciaacaGaaeqabaqabeGadaaakeaacqWGYbGCdaqhaaWcbaGafmyAaKMbauaacuWGQbGAgaqbaaqaaiabcIcaOiabd2gaTjabcMcaPaaaaaa@342B@ is the *C*_*α*_-*C*_*α *_distance between residues *i' *and *j' *in protein to that correspond to residues *i *and *j *in protein *n*. The variance is related to the sequence separation between residues *i *and *j*,σij2
 MathType@MTEF@5@5@+=feaafiart1ev1aaatCvAUfKttLearuWrP9MDH5MBPbIqV92AaeXatLxBI9gBaebbnrfifHhDYfgasaacH8akY=wiFfYdH8Gipec8Eeeu0xXdbba9frFj0=OqFfea0dXdd9vqai=hGuQ8kuc9pgc9s8qqaq=dirpe0xb9q8qiLsFr0=vr0=vr0dc8meaabaqaciaacaGaaeqabaqabeGadaaakeaaiiGacqWFdpWCdaqhaaWcbaGaemyAaKMaemOAaOgabaGaeGOmaidaaaaa@324D@ = |*i *- *j*|^0.15^, and the normalization is ℵ
 MathType@MTEF@5@5@+=feaafiart1ev1aaatCvAUfKttLearuWrP9MDH5MBPbIqV92AaeXatLxBI9gBaebbnrfifHhDYfgasaacH8akY=wiFfYdH8Gipec8Eeeu0xXdbba9frFj0=OqFfea0dXdd9vqai=hGuQ8kuc9pgc9s8qqaq=dirpe0xb9q8qiLsFr0=vr0=vr0dc8meaabaqaciaacaGaaeqabaqabeGadaaakeaacqGH1ecWaaa@2E4E@ = ((*N*_*seq*_* – *1) (*N*_*res*_* – k*))^-1^, where *N*_*seq *_is the number of proteins in the set, *N*_*res *_is the number of residues in protein *n*, and *k = *3 except when residue *i *is the N or C-terminus in which case *k = *2.

**Q**_H _– For measuring the similarity between two structures, we use Q_H_, which we have previously derived [21,20]. Like Q_res_, it is also adapted from Q, but accounts for the presence of insertions in the structure. Briefly, Q_H _calculates an overall score for the similarity of two structures by summing the similarity of all residues and then adding a term for each gap in the alignment. The more that an insertion perturbs the structure of nearby regions, the lower the resulting Q_H _value.  

**QR factorization** – The sequence and structure QR algorithms eliminate the redundancy from a collection of sequences or structures, respectively. The output is the smallest set of sequences or structures that represents the evolutionary diversity present in the initial group. These algorithms are based on a QR factorization with column pivoting of a matrix encoding the sequence or structure alignment. We have described each of these algorithms and their utility in developing EPs previously [21,22].

**STAMP** – The STAMP structural alignment program generates both structural superpositions and sequence alignments using tertiary structure comparisons [37]. Two modifications were made to the STAMP structural alignment program included with MultiSeq. First, the program was modified to work with RNA and DNA by allowing it to read structure files containing the phosphate backbone atoms of nucleic acid molecule and to recognize the residues contained in these files. Second, the program was modified to insert gaps into the multiple sequence alignment so that the trailing, poorly aligned ends of different structures will be gapped with respect to one another. These end-gaps are a natural result of the dynamic programming local alignment algorithm used by STAMP.

**C++ bioinformatics library** – Many of the algorithms are written in C++, since TCL is less suited for computationally intensive work. To facilitate the development and implementation of these algorithms, we have developed *libbiokit*, a bioinformatics toolkit. This library is comprised of classes that perform file I/O, such as FASTA and PDB readers and writers; classes that represent commonly used bioinformatic data structures, like sequence and structure alignments; and stand-alone utilities that execute the QR, Q_H_, Q_res_, and phylogenetic algorithms along with other standard measures used in the analysis of bioinformatic data. Libbiokit is packaged with MultiSeq and is also available separately as open source software from our website [62].

## Conclusion

MultiSeq allows new approaches to be taken in bioinformatic analysis: new relationships can be found and investigated by combining sequence and structure data; automatic download and use of metadata along with flexible grouping encourages organized analysis of unfamiliar data; the ability to remove redundancy from large sets of data helps to focus and speed up evolutionary analyses; and integration with several popular bioinformatics tools along with a versatile input and output ability reduce the time and "busy work" overhead of performing any analysis. MultiSeq extends VMD's capabilities into the realm of sequences based data and we hope that MultiSeq will help bring more widespread use of sequence data to the world of structural biology and vice versa.

MultiSeq is included with VMD starting with version 1.8.5 [69]. MultiSeq benefits from VMD's cross platform nature and currently runs on numerous operating systems, including Linux, Mac OS X, Solaris, and Windows. Metadata databases are automatically downloaded and updated via the Internet and can be stored either on the user's local machine or a workgroup file server. The use of BLAST for searching requires a locally installed version of the BLAST software from NCBI and sequence databases stored either on the local machine or a workgroup server. Detailed instructions on configuring the software are available in the MultiSeq manual available online [70]. A tutorial is also available from the NIH Resource for Macromolecular Modeling and Bioinformatics to assist in learning how to use the features of MultiSeq described in this article [52].

## Availability and requirements

• Project name: MultiSeq

• Project home page: 

• Operating System(s): Linux, Mac OS X, Unix, Windows

• Programming language: C++, TCL

• License: University of Illinois Open Source License, GNU LGPL

## Authors' contributions

ER, JE, and DW designed and developed the MultiSeq software. ZLS provided guidance on design and algorithm development. All authors contributed to drafting the manuscript.

## References

[B1] Berman HM, Westbrook J, Feng Z, Gilliland G, Bhat TN, Weissig H, Shindyalov IN, Bourne PE (2000). The Protein Data Bank. Nucleic Acids Res.

[B2] Andreeva A, Howorth D, Brenner SE, Hubbard TJP, Chothia C, Murzin AG (2004). SCOP database in 2004: refinements integrate structure and sequence family data. Nucleic Acids Res.

[B3] Murzin AG, Brenner SE, Hubbard T, Chothia C (1995). SCOP: a structural classification of proteins database for the investigation of sequences and structures. J Mol Biol.

[B4] Chandonia JM, Hon G, Walker NS, Lo Conte L, Koehl P, Levitt M, Brenner SE (2004). The ASTRAL Compendium in 2004. Nucleic Acids Res.

[B5] Orengo CA, Michie AD, Jones S, Jones DT, Swindells MB, Thornton JM (1997). CATH – a hierarchic classification of protein domain structures. Structure.

[B6] Berman HM, Olson WK, Beveridge DL, Westbrook J, Gelbin A, Demeny T, Hsieh SH, Srinivasan AR, Schneider B (1992). The nucleic acid database. A comprehensive relational database of three-dimensional structures of nucleic acids. Biophys J.

[B7] Pruitt KD, Tatusova T, Maglott DR (2005). NCBI Reference Sequence (RefSeq): a curated non-redundant sequence database of genomes, transcripts and proteins. Nucleic Acids Res.

[B8] Benson DA, Karsch-Mizrachi I, Lipman DJ, Ostell J, Wheeler DL (2005). GenBank. Nucleic Acids Res.

[B9] Cochrane G, Aldebert P, Althorpe N, Andersson M, Baker W, Baldwin A, Bates K, Bhattacharyya S, Browne P, van den Broek A, Castro M, Duggan K, Eberhardt R, Faruque N, Gamble J, Kanz C, Kulikova T, Lee C, Leinonen R, Lin Q, Lombard V, Lopez R, McHale M, McWilliam H, Mukherjee G, Nardone F, Pastor MPG, Sobhany S, Stoehr P, Tzouvara K, Vaughan R, Wu D, Zhu W, Apweiler R (2006). EMBL Nucleotide Sequence Database: developments in 2005. Nucleic Acids Res.

[B10] Okubo K, Sugawara H, Gojobori T, Tateno Y (2006). DDBJ in preparation for overview of research activities behind data submissions. Nucleic Acids Res.

[B11] Markowitz VM, Korzeniewski F, Palaniappan K, Szeto E, Werner G, Padki A, Zhao X, Dubchak I, Hugenholtz P, Anderson I, Lykidis A, Mavromatis K, Ivanova N, Kyrpides NC (2006). The integrated microbial genomes (IMG) system. Nucleic Acids Res.

[B12] Cole JR, Chai B, Farris RJ, Wang Q, Kulam SA, McGarrell DM, Garrity GM, Tiedje JM (2005). The Ribosomal Database Project (RDP-II): sequences and tools for high-throughput rRNA analysis. Nucleic Acids Res.

[B13] Boeckmann B, Bairoch A, Apweiler R, Blatter MC, Estreicher A, Gasteiger E, Martin MJ, Michoud K, O'Donovan C, Phan I, Pilbout S, Schneider M (2003). The SWISS-PROT protein knowledgebase and its supplement TrEMBL in 2003. Nucleic Acids Res.

[B14] Cannone JJ, Subramanian S, Schnare MN, Collett JR, D'Souza LM, Du Y, Feng B, Lin N, Madabusi LV, Muller KM, Pande N, Shang Z, Yu N, Gutell RR (2002). The comparative RNA web (CRW) site: an online database of comparative sequence and structure information for ribosomal, intron, and other RNAs. BMC Bioinformatics.

[B15] Sprinzl M, Horn C, Brown M, loudovitch A, Steinberg S (1998). Compilation of tRNA sequences and sequences of tRNA genes. Nucleic Acids Res.

[B16] Lowe TM, Eddy SR (1997). tRNAscan-SE: a program for improved detection of transfer RNA genes in genomic sequence. Nucleic Acids Res.

[B17] Woese CR, Olsen GJ, Ibba M, Soll D (2000). Aminoacyl-tRNA synthetases, the genetic code, and the evolutionary process. Microbiol Mol Biol Rev.

[B18] Socolich M, Lockless SW, Russ WP, Lee H, Gardner KH, Ranganathan R (2005). Evolutionary information for specifying a protein fold. Nature.

[B19] Chothia C, Lesk AM (1986). The relation between the divergence of sequence and structure in proteins. EMBO J.

[B20] O'Donoghue P, Luthey-Schulten Z (2003). On the evolution of structure in the aminocyl-tRNA synthetases. Microbiol Mol Bio Rev.

[B21] O'Donoghue P, Luthey-Schulten Z (2005). Evolutionary profiles derived from the QR factorization of multiple structural alignments gives an economy of information. J Mol Biol.

[B22] Sethi A, O'Donoghue P, Luthey-Schulten Z (2005). Evolutionary profiles from the QR factorization of multiple sequence alignments. Proc Natl Acad Sci USA.

[B23] O'Donoghue P, Sethi A, Woese CR, Luthey-Schulten ZA (2005). The evolutionary history of Cys-tRNA^Cys ^formation. Proc Natl Acad Sci USA.

[B24] Humphrey W, Dalke A, Schulten K (1996). VMD – Visual Molecular Dynamics. J Mol Graph.

[B25] Eargle J, Wright D, Luthey-Schulten Z (2006). Multiple Alignment of protein structures and sequences for VMD. Bioinformatics.

[B26] Cole JR, Chai B, Marsh TL, Farris RJ, Wang Q, Kulam SA, Chandra S, McGarrell DM, Schmidt TM, Garrity GM, Tiedje JM (2003). The Ribosomal Database Project (RDP-II): previewing a new autoaligner that allows regular updates and the new prokaryotic taxonomy. Nucleic Acids Res.

[B27] Parry-Smith DJ, Payne AW, Michie AD, Attwood TK (1998). CINEMA-a novel colour INteractive editor for multiple alignments. Gene.

[B28] Thompson JD, Gibson TJ, Plewniak F, Jeanmougin F, Higgins DG (1997). The CLUSTAL_X windows interface: flexible strategies for multiple sequence alignment aided by quality analysis tools. Nucleic Acids Res.

[B29] Clamp M, Cuff J, Searle SM, Barton GJ (2004). The Jalview Java alignment editor. Bioinformatics.

[B30] Thompson JD, Higgins DG, Gibson TJ (1994). CLUSTAL W: improving the sensitivity of progressive multiple sequence alignment through sequence weighting, position-specific gap penalties and weight matrix choice. Nucleic Acids Res.

[B31] Eddy SR (1998). Profile hidden Markov models. Bioinformatics.

[B32] Notredame C, Higgins DG, Heringa J (2000). T-Coffee: A novel method for fast and accurate multiple sequence alignment. J Mol Biol.

[B33] Johnson JM, Mason K, Moallemi C, Xi H, Somaroo S, Huang ES (2003). Protein family annotation in a multiple alignment viewer. Bioinformatics.

[B34] Altschul SF, Madden TL, Schaffer AA, Zhang J, Zhang Z, Miller W, Lipman DJ (1997). Gapped BLAST and PSI-BLAST: a new generation of protein database search programs. Nucleic Acids Res.

[B35] Kumar S, Tamura K, Nei M (2004). MEGA3: Integrated software for Molecular Evolutionary Genetics Analysis and sequence alignment. Brief Bioinform.

[B36] Sayle RA, Milner-White EJ (1995). RASMOL: biomolecular graphics for all. Trends Biochem Sci.

[B37] Russell RB, Barton GJ (1992). Multiple protein sequence alignment from tertiary structure comparison: assignment of global and residue confidence levels. Proteins.

[B38] Frishman D, Argos P (1995). Knowledge-based protein secondary structure assignment. Proteins.

[B39] Olson WK, Bansal M, Burley SK, Dickerson RE, Gerstein M, Harvey SC, Heinemann U, Lu XJ, Neidle S, Shakked Z, Sklenar H, Suzuki M, Tung CS, Westhof E, Wolberger C, Berman HM (2001). A standard reference frame for the description of nucleic acid base-pair geometry. J Mol Biol.

[B40] Guex N, Diemand A, Peitsch MC (1999). Protein modelling for all. Trends Biochem Sci.

[B41] Canutescu AA, Dunbrack RLJ (2005). MollDE: a homology modeling framework you can click with. Bioinformatics.

[B42] Canutescu AA, Shelenkov AA, Dunbrack RLJ (2003). A graph-theory algorithm for rapid protein side-chain prediction. Protein Sci.

[B43] Neshich G, Borro LC, Higa RH, Kuser PR, Yamagishi MEB, Franco EH, Krauchenco JN, Fileto R, Ribeiro AA, Bezerra GBP, Velludo TM, Jimenez TS, Furukawa N, Teshima H, Kitajima K, Bava A, Sarai A, Togawa RC, Mancini AL (2005). The Diamond STING server. Nucleic Acids Res.

[B44] Marti-Renom MA, Stuart AC, Fiser A, Sanchez R, Melo F, Sali A (2000). Comparative protein structure modeling of genes and genomes. Annu Rev Biophys Biomol Struct.

[B45] Pettersen EF, Goddard TD, Huang CC, Couch GS, Greenblatt DM, Meng EC, Ferrin TE (2004). UCSF Chimera-a visualization system for exploratory research and analysis. J Comput Chem.

[B46] Abyzov A, Errami M, Leslin CM, Ilyin VA (2005). Friend, an integrated analytical front-end application for bioinformatics. Bioinformatics.

[B47] Wang Y, Geer LY, Chappey C, Kans JA, Bryant SH (2000). Cn3D: sequence and structure views for Entrez. Trends Biochem Sci.

[B48] Gruber TR (1993). A translation approach to portable ontologies. Knowledge Acquisition.

[B49] The Gene Ontology Project. http://www.geneontology.org/.

[B50] Attwood TK, Miller CJ (2001). Which craft is best in bioinformatics?. Comput Chem.

[B51] Sethi A, Eargle J, O'Donoghue P, Pogorelov T, Amaro R, Luthey-Schulten Z (2004). Evolutionary profiles derived from QR factorization of multiple sequence and structural alignments. CASP6 Abstracts.

[B52] Roberts E, Eargle J, Wright D, Dhaliwal B, Sethi A, O'Donoghue P, Luthey-Schulten Z (2006). Evolution of Biomolecular Structure. http://www.scs.uiuc.edu/~schulten/tutorials/evolution.

[B53] Altschul SF, Gish W, Miller W, Myers EW, Lipman DJ (1990). Basic local alignment search tool. J Mol Biol.

[B54] Wheeler DL, Chappey C, Lash AE, Leipe DD, Madden TL, Schuler GD, Tatusova TA, Rapp BA (2000). Database resources of the National Center for Biotechnology Information. Nucleic Acids Res.

[B55] Wallace IM, Blackshields G, Higgins DG (2005). Multiple Sequence Alignments. Curr Opinion Struct Biol.

[B56] Al-Lazikani B, Sheinerman FB, Honig B (2001). Combining multiple structure and sequence alignments to improve sequence detection and alignment: Application to the SH2 domains of Janus kinases. Proc Natl Acad Sci USA.

[B57] Heath MT (2002). Scientific Computing: An Introductory Survey.

[B58] Sokal RR, Michener CD (1958). A statistical method for evaluating systematic relationships. Univ Kansas Sci Bull.

[B59] Felsenstein J (1981). Evolutionary trees from DNA sequences: a maximum likelihood approach. J Mol Evol.

[B60] Hollich V, Milchert L, Arvestad L, Sonnhammer ELL (2005). Assessment of protein distance measures and tree-building methods for phylogenetic tree reconstruction. Mol Biol Evol.

[B61] Tufte E (1983). The Visual Display of Quantitative Information.

[B62] Luthey-Schulten Group Software. http://www.scs.uiuc.edu/~schulten/software.html.

[B63] Limbach PA, Crain PF, McCloskey JA (1994). Summary: the Modified Nucleosides of RNA. Nucl Acids Res.

[B64] Swofford DL (1998). PAUP*: phylogenetic analysis using parsimony (*and other methods).

[B65] Felsenstein J (2005). PHYLIP (Phylogeny Inference Package) version 36 Distributed by the author.

[B66] Guindon S, Gascuel O (2003). A simple, fast, and accurate algorithm to estimate large phylogenies by maximum likelihood. Syst Biol.

[B67] Ronquist F, Huelsenbeck JP (2003). MrBayes 3: Bayesian phylogenetic inference under mixed models. Bioinformatics.

[B68] Eastwood MP, Hardin C, Luthey-Schulten Z, Wolynes PG (2001). Evaluating protein structure-prediction schemes using energy landscape theory. IBM J Res Dev.

[B69] VMD – Visual Molecular Dynamics. http://www.ks.uiuc.edu/Research/vmd/.

[B70] MultiSeq – A Unified Bioinformatics Analysis Environment. http://www.scs.uiuc.edu/~schulten/multiseq.

